# Measurable Residual Disease-Guided Treatment to Prevent Relapse in Acute Myeloid Leukemia and Myelodysplastic Syndrome

**DOI:** 10.3389/fonc.2020.576924

**Published:** 2020-10-22

**Authors:** Jonathan Pan, Daniel Altman, Lindsay Wilde

**Affiliations:** Department of Medical Oncology, Thomas Jefferson University Hospital, Philadelphia, PA, United States

**Keywords:** acute myeloid leukemia, myelodysplastic syndrome, measurable residual disease, hypomethylating agents, azacitidine, stem-cell transplant

## Introduction

In patients with intermediate or poor risk acute myeloid leukemia (AML) or advanced myelodysplastic syndrome (MDS), allogeneic hematopoietic stem cell transplantation (ASCT) remains the only curative treatment (1). Although overall survival (OS) of these patients has improved over the past few decades, relapse rates remain high (2, 3). Following ASCT, up to 50% of patients will relapse, with the majority occurring the first 48 months (4, 5). The prognosis of patients who relapse is poor, with less than 20% surviving 2 years (6). Risk factors for relapse include reduced intensity conditioning-regimen, absence of graft-versus-host disease (GVHD), loss of donor chimerism, and the presence of measurable residual disease (MRD) (5, 7). Currently utilized methods for MRD detection include multi-parametric flow cytometry, quantitative polymerase chain reaction, and next-generation sequencing (8, 9). MRD detection is an important prognostic tool as MRD positivity portends a higher risk of relapse (10). Patients treated for AML who achieve morphologic complete remission (CR) but have MRD positivity at the time of ASCT have higher relapse rates and worse OS than MRD-negative patients (11). Similarly, patients with AML who develop MRD positivity after ASCT have a higher incidence of relapse and worse OS compared to those who remain MRD-negative (12). Given the poor prognosis of AML patients who become MRD-positive, strategies to prevent relapse are needed. Herein, we present arguments for and against the use of hypomethylating agents (HMA) to prevent morphologic AML/MDS relapse in this patient population. This review will focus on the recently published RELAZA-2 trial, one of the largest studies to investigate pre-emptive MRD-guided treatment using HMA (13).

RELAZA-2 was a German-multicentered, open-label, single-arm, phase II study assessing the efficacy of azacitidine in adults with MDS or AML who achieved morphologic CR after conventional chemotherapy or ASCT but developed MRD positivity within 24 months of treatment. MRD was detected by ≤80% CD34+ donor chimerism in ASCT patients, or an increase in NPM1 mutation or fusion genes including DEK-NUP214, RUNX1-RUNX1T1, or CBFb-MYH11, above 1% in the blood or bone marrow without concurrent hematologic relapse. Patients screened using CD34+ donor chimerism were monitored monthly for the first 6 months, followed by every 3 months until month 24. Patients screened using NPM1 or fusion genes had either bone marrow assessment every 3 months or peripheral blood assessment monthly. In patients who became MRD-positive, azacitidine 75mg/m^2^ days 1-7 was given every 29 days for 24 cycles. MRD was monitored after 6 cycles, with MRD-negative patients eligible for treatment de-escalation. The primary endpoint was relapse-free survival at 6 months after starting azacitidine.

Of the 198 patients screened, 53 (AML, n = 48; MDS, n = 5; prior treatment: chemotherapy, n = 29; ASCT, n = 24) became MRD-positive and were eligible for treatment. Median follow up was 13 months after the start of MRD-guided therapy. After 6 months of azacitidine, 31 patients (58%, 95% CI 44-72) were relapse free and alive (p < 0.0001; one-sided binomial test for null hypothesis p_ex_p < 0.3). 19 of the 31 patients achieved MRD-negativity; the other 12 patients were MRD-positive but were without hematologic relapse. At 12 months, relapse-free survival (RFS) was 46%, and the OS was 75%. Thirteen patients died: 10 from relapse and 3 from infection (1 possibly related to azacitidine). The most common high-grade adverse event was neutropenia which occurred in 42 patients (79%). Of the 198 patients who remained MRD-negative (n = 138), 12-month RFS was 88% and 12-month OS was 91%.

Among the ASCT patients (n = 24), 10 were on systemic immunosuppression at the time of treatment initiation. Four patients without a history of GVHD developed GVHD during azacitidine treatment. Two received concomitant donor-lymphocyte infusion (DLI) and 4 were on systemic immunosuppression 6 months after starting MRD-guided treatment. Out of the 24 ASCT patients, 17 (71%) were relapse-free at 6 months with 13 of the 17 achieving MRD-negativity.

## Point: The Case for Azacitidine in MRD-Positive Disease

There is sufficient evidence that MRD positivity yields a worse prognosis for patients with intermediate and poor-risk AML or MDS after chemotherapy or ASCT (10–12). Prior to the RELAZA-2 trial, the single-center, phase II Relaza-1 trial investigated pre-emptive treatment of 20 patients with poor-risk AML or MDS who were MRD-positive after ASCT (14). MRD positivity in this group was defined as CD34+ donor chimerism less than 80%. These patients were treated with at least 4 cycles of azacitidine. Ultimately, 16 patients responded, with a majority achieving the goal of CD34+ donor chimerism greater than 80%. Thirteen of the 20 patients eventually developed hematological relapse, however, the median time to relapse (TTR) was 231 days which was significantly delayed compared to historical controls.

Relaza-2 sought to confirm these results and demonstrated clear improvement in RFS after detection of MRD positivity (13). Median TTR was 422 days vs. historic trials demonstrating median TTR at 126, 255, and 61 days, respectively (12, 15, 16). Over one-third of the patients displayed a major MRD response. This group of patients had significantly better outcomes than patients with lesser responses. In fact, at 23 months, more than 60% of them were alive and relapse free. Azacitidine was well tolerated, with few significant adverse effects. As expected, neutropenia rates were high; however, rates of neutropenic fever remained low. Additionally, this study reinforced the importance of achieving MRD negativity in preventing disease relapse in this patient population.

Other studies have investigated HMA maintenance therapy following ASCT in patients with AML and MDS. An early phase trial demonstrated that low-dose decitabine maintenance was well tolerated in patients 50–100 days following ASCT, without a major impact on GVHD incidence (17). Preliminary results comparing low-dose azacitidine maintenance vs. observation after ASCT showed decrease in relapse rates (25.8 vs. 66.7%), increase in TTR (not reached vs. 4.1 months; p < 0.001), and improvement in OS (27.5 months vs. 7.6 months; p < 0.0001) favoring the azacitadine group (18). Azacitidine did not appear to increase the risk of GVHD in this study. These results suggest that HMAs are both safe and effective in the post-ASCT setting.

Patients with intermediate or poor-risk AML or MDS who are MRD-positive after chemotherapy or ASCT have unacceptably high rates of relapse and death. Further therapies to achieve MRD negativity in this setting are indicated. Maintenance azacitadine has shown benefit in improving RFS, delaying hematologic relapse with a tolerable side effect profile and should be a consideration in this patient population.

## Counterpoint: The Case Against Azacitidine in MRD-Positive Disease

Although RELAZA-2 demonstrated improved RFS at 6 months, this was a non-randomized phase II study without a control group and had a small, heterogeneous patient population including those previously treated with either chemotherapy alone or ASCT. Since this study combined both treatment groups, it is difficult to generalize the outcomes as MRD-positive patients post-ASCT are not the same population as the MRD-positive patients post-chemotherapy. Additionally, post-hoc analysis comparing outcomes between MRD-responders and non-responders showed there was no difference in OS at 6 months (HR, 0.4; 95% CI: 0.1–1.3, p = 0.112). The methodology used for MRD monitoring in this study, specifically using CD34+ donor chimerism of less than 80% in post-ASCT patients, has not been verified as a standard approach. There were also two post-ASCT patients who received a concomitant DLI which may have augmented treatment outcomes.

The complications of maintenance with azacitidine should also be taken into consideration, specifically the increase in hematologic toxicity and severe infection risk observed in Relaza-2 and a recent study investigating post-ASCT maintenance decitabine (17). In addition, although there is evidence that HMAs may immunologically mitigate GVHD while preserving the graft-versus-leukemia effect (19), there were four patients in the RELAZA-2 study who developed GVHD after azacitidine who had no prior history GVHD. Further studies are needed to assess the immunomodulatory impact of HMA in the post ASCT period.

In the era of cost-conscious health care, the financial burden of monthly azacitidine infusions should also be weighed against the overall benefit of treatment. Similarly, treatment impact on patient quality of life should be considered given frequent office visits and need for repeated bone marrow assessment to monitor MRD response.

It is also unclear if standard azacitidine is the optimal maintenance treatment of choice in this setting. CC-486, a novel oral formulation of azacitidine, showed good tolerability and low rate of relapse in an early phase I/II study in AML patients who achieved CR following ASCT (20). More recently, the phase III randomized, double blinded QUAZAR AML-001 trial investigated CC-486 maintenance in AML patients in first CR who are not candidate for curative consolidation such as ASCT. Results comparing the CC-486 to placebo groups showed significant improvements in OS (24.7 vs. 14.8 months; HR = 0.69, 95% CI: 0.55–0.86, p = 0.0009) and RFS (10.2 vs. 4.8 months; HR = 0.65, 95% CI: 0.52–0.81, p = 0.0001) (21). MRD status is being monitored in this study but those results have not yet been reported. Based on this study, CC-486 was recently approved by the Food and Drug Administration as maintenance treatment for AML patients in first CR who are unable to proceed to intensive consolidation therapy. Ongoing studies investigating AML maintenance treatment with azacitidine in combination with venetoclax and pembrolizumab, respectively, are being conducted and will evaluate the impact on MRD status [NCT03769532 and NCT04062266]. These studies may provide further insight on the optimal strategy in MRD-guided treatment of AML.

## Discussion

Given the dismal outcomes in relapsed AML and MDS, treatment of patients with MRD-positive disease may be an effective option to prevent relapse. The optimal management of these patients is still being evaluated but promising results were seen in the Relaza-2 trial which showed improvement in RFS and TTR using azacitidine in MRD-positive patients. The Relaza-2 authors hypothesized that the proposed mechanism of HMA in the ASCT group is immunologic induction of regulatory T-cells that mitigate GVHD while preserving the graft-versus leukemia effect, which has also been supported by pre-clinical data (22, 23). Questions that remain unanswered include the effect of HMA on ASCT complications, including GVHD, and whether the treatment toxicities, both physical and financial, are worth the overall benefit. Given the lack of alternative therapies and poor outcomes in this patient population, we would recommend consideration of maintenance azacitidine which appears effective in preventing frank relapse of AML and MDS. Future studies that could provide more insight on MRD-guided therapy include evaluating maintenance treatment in more homogenous patient populations such as separating ASCT patients from those who received chemotherapy alone. Investigating different HMA dosing levels and schedules, as well as alternate HMA formulations such as CC-486, may improve treatment efficacy, decrease toxicity, and ultimately decrease treatment burden. Methods for testing MRD could also be evaluated to determine the optimal strategy for MRD monitoring. Combining HMA with novel agents could yield a more effective maintenance. Ongoing randomized trials will hopefully provide further insight and help identify the optimal treatment strategy moving forward.

## Author Contributions

JP and DA conducted research and drafted and revised this article. LW provided critical revision and final editing support. All authors contributed to the article and approved the submitted version.

## Conflict of Interest

The handling editor is currently organizing a Research Topic with one of the authors [LW].

The authors declare that the research was conducted in the absence of any commercial or financial relationships that could be construed as a potential conflict of interest.
